# Glutamic acid promotes monacolin K production and monacolin K biosynthetic gene cluster expression in *Monascus*

**DOI:** 10.1186/s13568-016-0311-z

**Published:** 2017-01-10

**Authors:** Chan Zhang, Jian Liang, Le Yang, Shiyuan Chai, Chenxi Zhang, Baoguo Sun, Chengtao Wang

**Affiliations:** 1 Beijing Advanced Innovation Center for Food Nutrition and Human Health, Beijing Technology & Business University (BTBU), Beijing, 100048 China; 2 School of Food and Chemical Engineering, Beijing Technology & Business University, Beijing, 100048 China; 3 Beijing Engineering and Technology Research Center of Food Additives, Beijing Technology & Business University, Beijing, 100048 China; 4 Beijing Technology & Business University, Fucheng Road 11, Haidian District, Beijing, People’s Republic of China

**Keywords:** Gene expression, Glutamic acid, Monacolin K, *Monascus* RT-qPCR

## Abstract

This study investigated the effects of glutamic acid on production of monacolin K and expression of the monacolin K biosynthetic gene cluster. When *Monascus* M1 was grown in glutamic medium instead of in the original medium, monacolin K production increased from 48.4 to 215.4 mg l^−1^, monacolin K production increased by 3.5 times. Glutamic acid enhanced monacolin K production by upregulating the expression of *mokB*-*mokI*; on day 8, the expression level of *mokA* tended to decrease by Reverse Transcription-polymerase Chain Reaction. Our findings demonstrated that *mokA* was not a key gene responsible for the quantity of monacolin K production in the presence of glutamic acid. Observation of *Monascus* mycelium morphology using Scanning Electron Microscope showed glutamic acid significantly increased the content of *Monascus* mycelium, altered the permeability of *Monascus* mycelium, enhanced secretion of monacolin K from the cell, and reduced the monacolin K content in *Monascus* mycelium, thereby enhancing monacolin K production.

## Introduction

*Monascus* species, which are characteristically found in East Asian countries, have been influential in local life and culture, and have received attention worldwide because of their diverse products (Cheng et al. 2016) and abundant beneficial metabolites (Ming-Jen et al. 2010). *Monascus* species are known to produce various secondary metabolites with polyketide structures, including monacolins (Ming-Tao et al. 2013), pigments (Dajung et al. 2014), γ-aminobutyric acid (Su et al. 2003), and citrinins (Radu et al. 2012a; Zhang et al. 2016).

Monacolin K, an inhibitor of cholesterol biosynthesis, was the first monacolin isolated from the cultures of *Monascus ruber*. This compound has also been independently found in *Aspergillus terreus,* in which it is designated lovastatin (Nezami et al. 2012). Monacolin K is able to act on cholesterol biosynthesis (Radu et al. 2012b) and can block the activity of HMG-CoA reductase as a competitive inhibitor (Suzuki and Imai 2010). Moreover, monacolin K is thought to have wide uses in the clinical setting. For example, monacolin K is one of the most effective drugs available for the treatment of hyperlipidemia (Feuerstein and Bjerke 2012). Monacolin K can reduce the expression of pro-inflammatory transcription factors, lower the extent of atherosclerosis, and promote apotosis in malignant thyroid cells (Chen et al. 2014). In addition, monacolin K has the ability to inhibit breast cancer cell proliferation (Patel 2016).

Monacolin K can be produced by *Monascus* during liquid or solid fermentation (Yu et al. 2013). The general production of monacolin K in liquid fermentation is lower (5-130 mg/L) than that in solid fermentation. However, the liquid fermentation process is relatively simpler than the solid fermentation process (Vendruscolo et al. 2016). Moreover, Previous studies have shown that the secondary metabolites of *Monascus* fermentation are strongly influenced by the environmental factors (Kang et al. 2013a; Rashmi and Padmavathi 2014), such as particularly nitrogen sources (Vendruscolo et al. 2016), can influence the production of secondary metabolites in *Monascus*. However, the effects of amino acids on the secondary metabolites of *Monascus* are multifaceted, the impact mechanisms are till unclear.

**Fig. 1:**
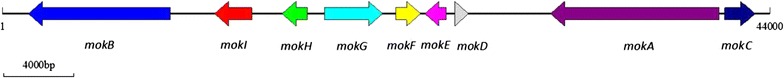
Map of the monacolin K biosynthetic gene clusters. *Arrows* show the genes and directions of transcription

The monacolin K biosynthetic gene cluster (Sakai et al. 2009) has been identified in previous studies (Fig. 1). According to the similarities with lovastatin synthetic genes (LNKS) in *Aspergillus*, researchers have identified nine genes (*mokA*-*mokI*) associated with the monacolin K synthesis and have proposed the functions of these genes (Chen et al. 2008). Each gene has different functions, and they are all important to the synthesis of monacolin K, i.e., *mokA* (polyketide synthase), *mokB* (polyketide synthase), *mokC* (P450 monooxygenase), *mokD* (oxidoreductase), *mokE* (dehydrogenase), *mokF* (transesterase), *mokG* (HMG-CoA reductase), *mokH* (transcription factor) and *mokI* (efflux pump).

In the study, we analyzed the effects of glutamic acid as a nitrogen source on the complex regulation of gene expression for monacolin K synthesis. We further assessed the influence of glutamic acid on mycelium content, pH, mycelium morphology, and monacolin K production in liquid culture medium, which are main factors to monacolin K production in *Monascus*.

## Materials and methods

### Fungal strain and culture conditions

*Monascus* M1 was obtained from The Chinese General Microbiological Culture Collection Center (Strain Number, CGMCC 3.0568), China. *Monascus* M1, which is a stable producer of monacolin K, was maintained on potato/dextrose/agar (PDA) for 5 days at 30 °C. All *Monascus* were cultured with 50 ml seed medium containing (per liter): 30 g glucose, 15 g Soybean powder, 1 g MgSO_4_·7H_2_O, 2 g KH_2_PO_4_, 70 g glycerol, 2 g NaNO_3_,and 10 g peptone at a neutral pH. The cultures were incubated at 30 °C for 48 h with shaking at 200 rpm. For monacolin K production and gene expression testing, 5 ml of the seed medium was inoculated into two types of fermentation medium (50 ml). The original fermentation medium contained (per liter): 20 g rice powder, 1 g MgSO_4_·7H_2_O, 2 g ZnSO_4_·7H_2_O, 2.5 g KH_2_PO_4_, 90 g glycerol, 5 g NaNO_3_ and 10 g peptone at a neutral pH. The glutamic acid fermentation medium contained all of the above components of the original fermentation medium plus 10 mM glutamic acid. The cultures were incubated at 30 °C for 48 h with shaking at 150 rpm, followed by incubation at 25 °C for 240 h with shaking at 150 rpm.

### Analysis of *Monascus* content and pH

For analysis of *Monascus* content, 5 ml liquid fermentation medium was placed on four layers of gauze for filtering, and the gauze was then washed with sterile water until the liquid was colorless. The gauze was dried in an oven at 60 °C overnight and then weighed. The pH value was determined in different media using a pH meter. The experiment was performed in triplicate, and values are presented as average values of three independent measurements.

### Analysis of monacolin K production

For analysis of monacolin K production, 5 ml of the fermentation medium was inoculated into 15 ml of 75% methanol (v/v), sonicated for 10 min, and allowed to settle over night. Monacolin K production was determined by high-performance liquid chromatography (HPLC) on a C18 column at 25 °C (5 μm, 150 × 4.6 mm) after filtration of the supernatant through a 0.45 μm filter. The mobile phase was 0.1% H_3_PO_4_/methanol (1:3, v/v), running at 1 ml min^−1^. The eluate was monitored by ultraviolet spectroscopy at a wavelength of 237 nm. Monacolin K from the National Institutes for Food and Drug Control in China was used as the standard.

### Quantitative real-time reverse transcription polymerase chain reaction (RT-qPCR) analysis of monacolin K biosynthetic gene clusters

*Monascus* mycelia were harvested from the monacolin K production medium and stored in liquid nitrogen for RNA extraction. Total RNA was extracted from mycelia using an RNAprep Pure Plant Kit (Tiangen-bio, Beijing, China) according to the manufacturer’s protocol. First-strand cDNA was synthesized using a FastQuant RT Kit (with gDNase) (Tiangen-bio, Beijing, China), with the FQ-RT Primer Mix. Gene expression was monitored by RT-qPCR, carried out using SYBR Green PCR master mix (Tiangen-bio, Beijing, China). Primers for *mokA, mokB, mokC*, *mokD, mokE, mokF*, *mokG, mokH, mokI* (NCBI accession No. DQ176595.1) and *GAPDH* genes (NCBI accession No. HQ123044.1) were designed by Beacon Designer8 (Table 1).

**Table 1:** Primer pairs used for amplification of the monacolin K gene cluster in *Monascus*

Genes	Primer pairs	Length (bp)	Tm value
*mokA*-F	5′- GACCTCGGTCATCTTGGC -3′	18	59.1
*mokA*-R	5′- TTGTTCCAAGCGGTCTTC -3′	18	57.3
*mokB*-F	5′- AAACATCGTCACCAGTCT -3′	18	50.7
*mokB*-R	5′- CTAAGTCGGGCATCTACC -3′	18	53.1
*mokC*-F	5′- CAAGCTGCGAAATACACCAAGCCTC -3′	25	63.6
*mokC*-R	5′- AGCCGTGTGCCATTCCTTGTTGTCC -3′	25	65.3
*mokD*-F	5′- TTCATCTGCTGCTGGTAT -3′	18	59.8
*mokD*-R	5′- AACTTCTCACCGTCAATG -3′	18	58.7
*mokE*-F	5′- ATCGCAGGTCACGCACATCCAAGTC -3′	25	72.3
*mokE*-R	5′- GTAAAGGCAGCCCGAGCAGCTTCAT -3′	25	71.1
*mokF*-F	5′- GAGATCATAGTGGCCGACTGAA -3′	22	59.8
*mokF*-R	5′- ACCGTCTCATCCAACCTCACGA -3′	22	56.1
*mokG*-F	5′- CCAGGTAACCAACGGATTA -3′	19	56
*mokG*-R	5′- GATCAGAGCAGTCACCAG -3′	18	52
*mokH*-F	5′- CAGGAAATCTGGACTTACCCCATTG -3′	25	65.8
*mokH*-R	5′- TGTTGGATTGTTGTTGGAGATATAC -3′	25	59.2
*mokI*-F	5′- ATGTTGAATGGCAATGATGG -3′	20	60.9
*mokI*-R	5′- CAGCGTGGGTGATGTATC -3′	18	61.7
*GAPDH*-F	5′- CCGTATTGTCTTCCGTAAC -3′	19	55.4
*GAPDH*-R	5′- GTGGGTGCTGTCATACTTG -3′	19	57.6

RT-qPCR was performed to determine the expression patterns of *Monascus* M1 in different fermentation culture media using a CFX96 Real-Time PCR Detection System version with 3.0 software (Bio-Rad, Hercules, CA, USA). After reverse transcription of total RNA into cDNA. cDNA was then used for qPCR with unigene-specific primers. The amplification program was as follows: 95 °C for 15 min, followed by 40 cycles of 95 °C for 10 s, 60 °C for 30 s and 72 °C for 30 s. Amplification was performed using SuperReal PreMix Plus (SYBR Green) for the fluorophore SYBR green with fluorescein. The relative abundance of transcripts was calculated by the comparative threshold cycle (CT) method. *GAPDH* was used as the housekeeping reference gene. RT-qPCR was carried out in triplicate for each sample.

### Scanning electron microscopy (SEM) analysis of *Monascus* mycelium

Scanning electron microscopy was used to observe the morphological differences in mycelia for in different media. M1 mycelium cells were fixed in 25% glutaraldehyde solution in phosphate-buffered sline (PBS) for 12 h at 25 °C temperature. Mycelium cell suspensions were rinsed with 0.1 M H_3_PO_4_ solution in PBS (pH7.2) and harvested by centrifugation (12,000 rpm, 5 min, 4 °C). The supernatants were removed, and the mycelia were resuspended, dehydrated using a graded ethanol series (30, 50, 70, 80, 90 and 100%), and the mycelia were harvested by centrifugation (12,000 rpm, 5 min). The ethanol was removed with isoamyl acetate-ethanol solution (1:1, v:v), incubated for 10 min, and centrifuged at 12,000 rpm for 5 min. The mycelia were resuspended in a suitable amount of hexamethyl disilazane (HMDS), with cotton wool plugging the upper part of the tube during centrifugation, and the sample was dried to a powder at 60 °C. After primary fixation, the mycelia were coated with gold–palladium for 2 min. Photomicrographs were then acquired using a VEGA 3LMU/LMH scanning electron microscope (TESCAN, Brno, Czech republic).

## Results

### Effects of glutamic acid on monacolin K production and biomass

**Fig. 2:**
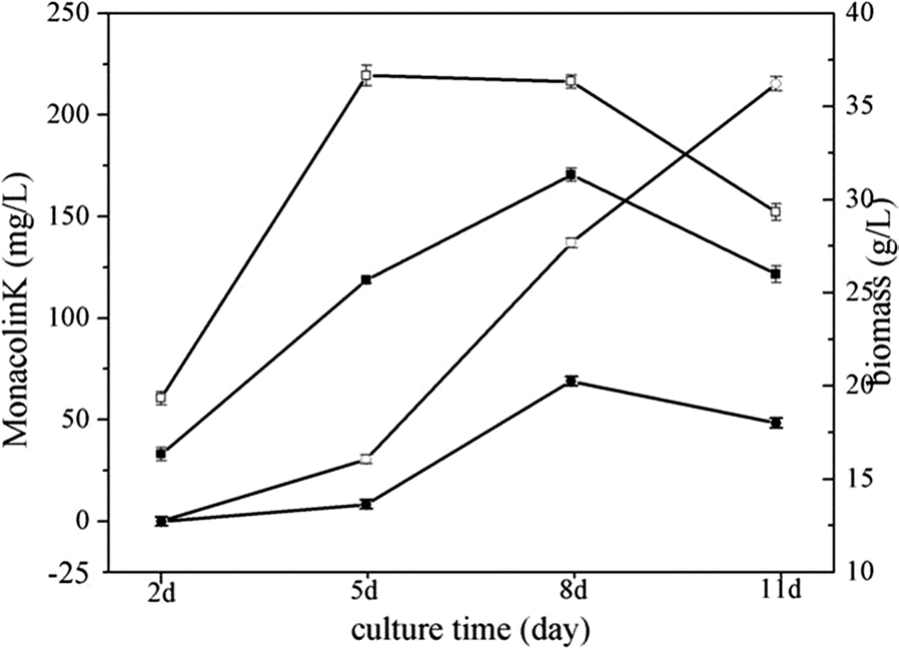
Effects of glutamic acid on monacolin K production and biomass. Monacolin K content in *Monascus* M1 grown in original medium (*filled circle*) or glutamic acid-containing medium (*open circle*), as assessed by HPLC. Biomass content of *Monascus* M1 grown in original medium (*filled square*) or glutamic acid-containing medium (*open square*), as assessed by weight. Samples were collected every 3rd day from days 2–11. Additionally, 500 mg (wet weight) of mycelia was weighed for RNA extraction, and the rest was used for determining the biomass. Biomass in original medium and glutamic acid-containing medium was estimated by determining the dry weight of the mycelia. Values are the average of three independent experiments. *Error bars* represent the standard deviation (n = 3)

**Fig. 3:**
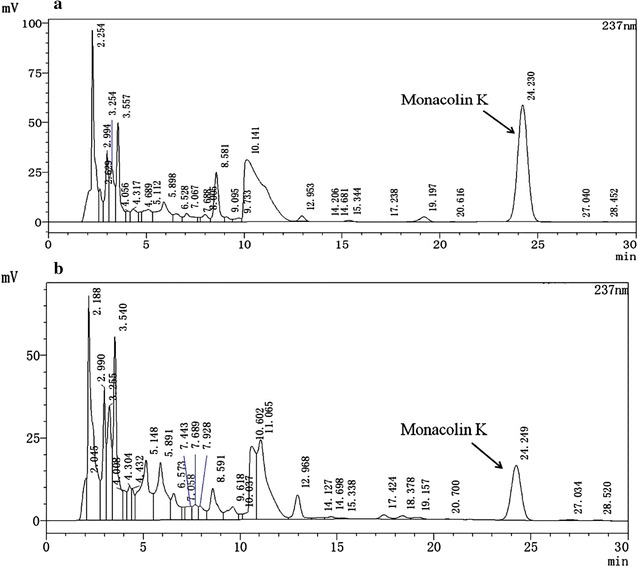
The maximal production of monacolin K was observed on day 11 in glutamic acid medium (**a**) and on day 8 in the original medium (**b**) by HPLC

At the beginning of the study, we constructed growth curves and determined the four key stages of *Monascus* M1 growth and the most suitable amount of glutamic acid. This analysis showed that the adjustment phase, logarithmic phase, and stabilization stage of M1 occurred on days 2, 5 and 11, respectively. The maximum monacolin K biosynthesis quality was achieved on day 8. A number of pre experimental results showed that the amount of 10 mM glutamic acid and the time of initial fermentation is the most optimal program of promoting the monacolin K production in *Monascus*. Figure 2 shows accumulation of monacolin K content from *Monascus* M1 in the original medium and glutamic acid-containing medium. In glutamic acid medium, maximal production of monacolin K (215.4 mg l^−1^) was observed on day 11 by HPLC (Fig. 3a). In the original medium, maximal monacolin K production (68.6 mg l^−1^) was observed on day 8 by HPLC (Fig. 3b). Glutamic acid was found to substantially alter the biomass of *Monascus* M1. For example, in glutamic acid medium, the maximal biomass (36.7 mg l^−1^) was found on day 5, whereas that in the original medium (31.3 mg l^−1^) was found on the day 8.

In addition, we tracked changes in pH values in different culture media. Table 2 shows changes in pH values in different culture media. In the adjustment and logarithmic phases (on days 2 and 5, respectively) of *Monascus* M1, the pH value of the glutamic acid fermentation medium was generally low. During the stabilization stage (on day 8) and the monacolin K biosynthesis quality maximum stage, minor changes in the pH values of the different culture media were observed.

**Table 2:** Change of pH values in different culture medium

pH value of fermentation broth	Time (d)			
	2	5	8	11
Original medium	5.15	6.02	5.89	5.55
Glutamic acid medium	4.68	4.88	6.06	5.74

### Effects of glutamic acid on the mycelial morphlogy of *Monascus*

**Fig. 4:**
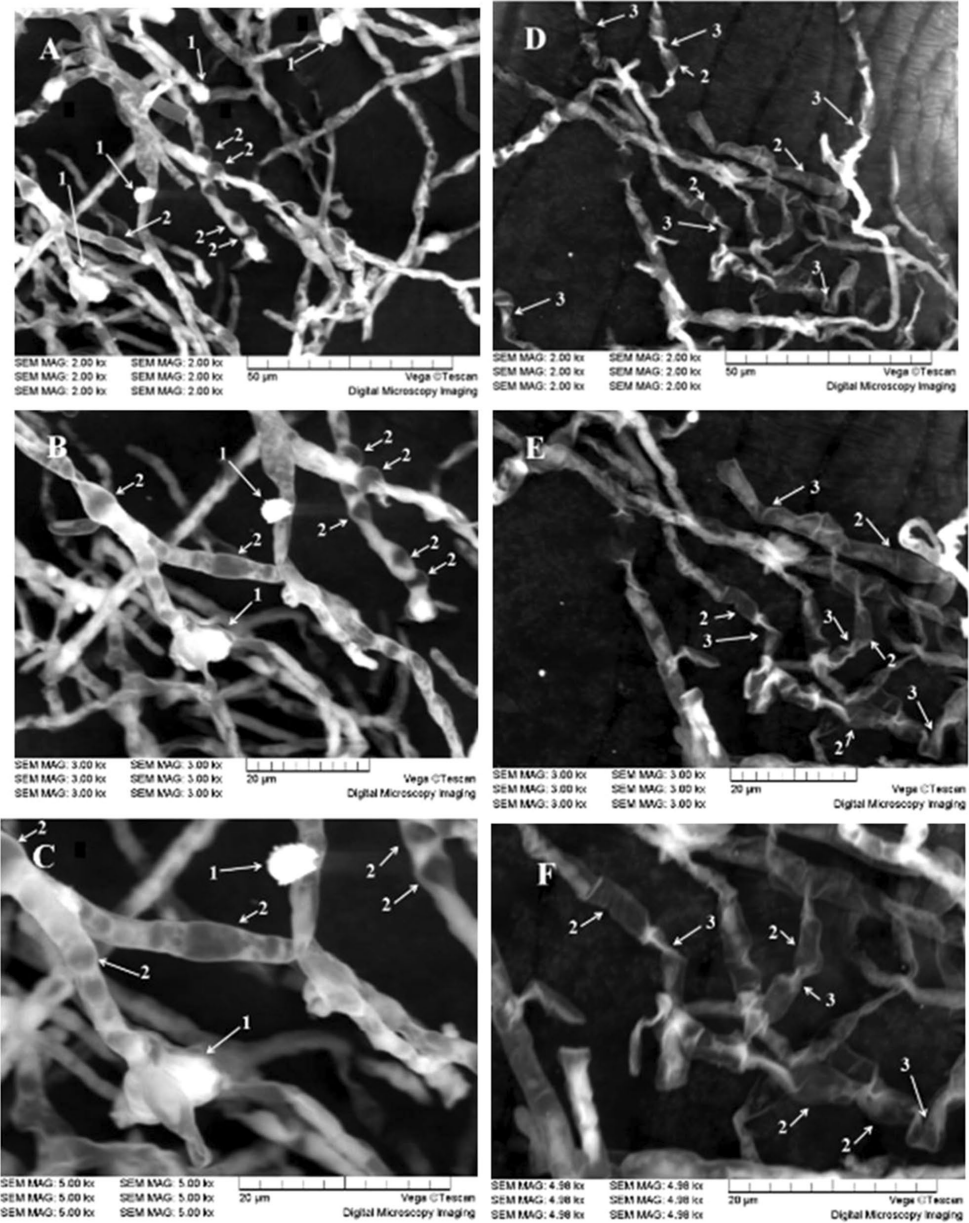
Morphology (**A**) of *Monascus* M1 in different culture medium with different magnifications factor, 2000× , 3000× and 5000× , respectively. M1 was cultured in original medium (**A**–**C**), glutamic medium (**D**–**F**) for 11 days at 25 °C with shaking at 150 rpm. Spore (*arrows #1*), Bulges (*arrows #2*) and Folds (*arrows #3*) were visible

As shown in the SEM images in Fig. 4, compared with the control group (mycelia in original culture medium), the mycelia morphology in glutamic acid-containing culture medium exhibited more folds and larger bulges, forming a variety of oddly shaped mycelia. In contrast, the radius of M1 mycelia in the original culture medium was uniform, and the top branches of the mycelia were formed of a single or multiple conidia, with spherical or ellipsoidal spores. Compared with the mycelia in original culture medium, those in glutamic acid-containing medium exhibited more prominent bulges, suggesting that the permeability of the mycelia may be enhanced.

Therefore, we speculated that glutamate could improve the permeability of mycelia. Furthermore, secretion of monacolin K outside of the cell was enhanced, monacolin K content in the cell was reduced, and monacolin K production was improved in glutamic acid-containing medium.

### Effects of glutamic acid on the expression of monacolin K biosynthetic genes

**Fig. 5:**
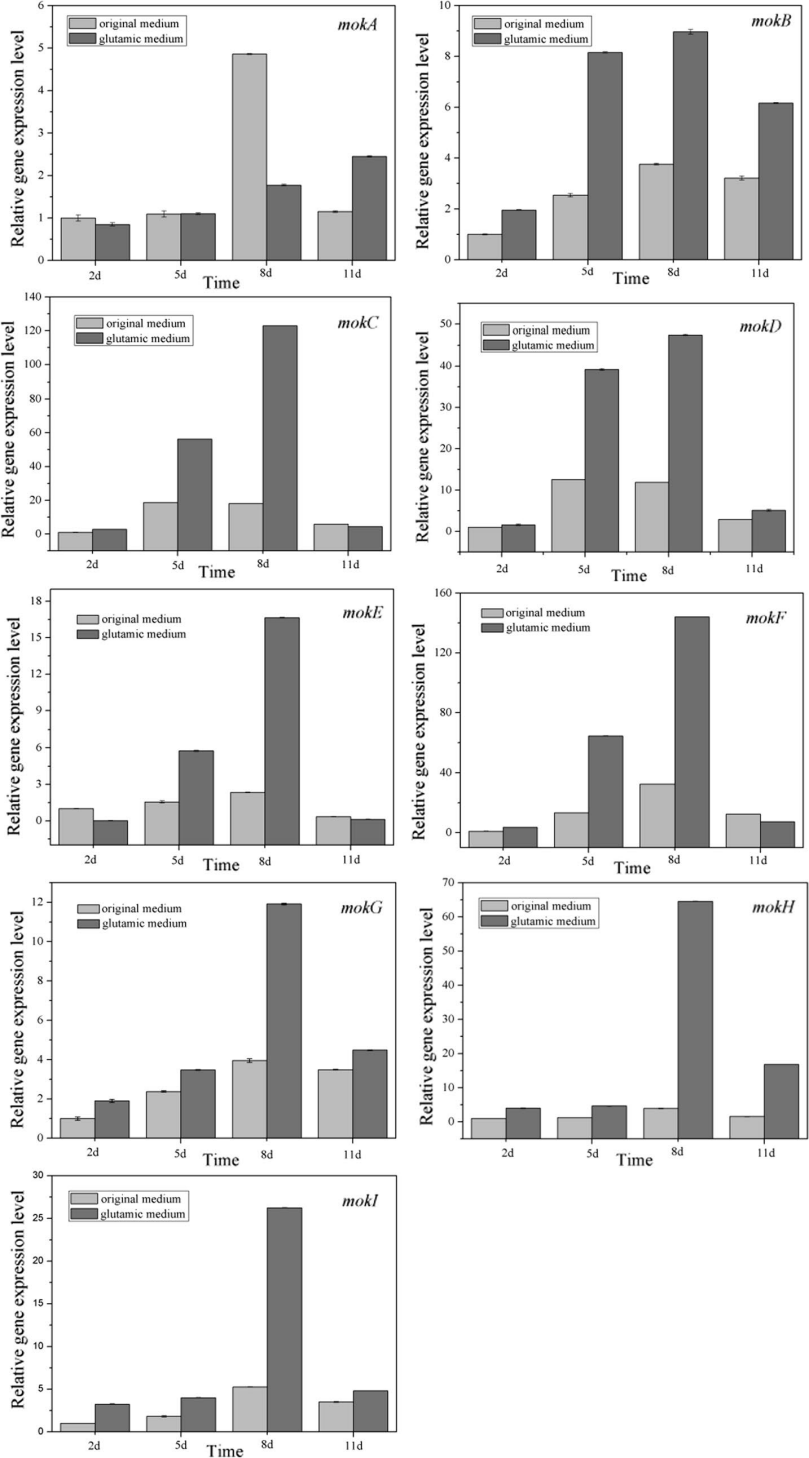
Expression of monacolin K biosynthesis-related genes during fermentation. Gene expression for various monacolin K biosynthesis-related genes was analyzed by RT-qPCR. Test samples corresponded one-to-one with samples used for monacolin K testing. Transcript levels were normalized to that of the *GAPDH* gene. The mRNA expression levels on day 2 in original medium were used as reference values (value: 1.0). Data are expressed as the relative mRNA level for each gene and represent the average values from three separate experiments. *Error bars* represent the standard deviation (n = 3). ***P < 0.001, *P < 0.05 compared with mRNA ratio in glutamic acid-containing medium

Figure 5 shows the expression profiles of monacolin K biosynthetic genes in original medium and glutamic acid-containing medium. The expression levels of *mokA*, *mokB*, *mokC*, *mokD*, *mokE*, *mokF*, *mokG*, *mokH*, and *mokI* were positively correlate with monacolin K accumulation when cultured in original medium. On day 8, gene expression and monacolin K production reached a maximum in original medium. A similar trend was observed in glutamic acid-containing medium; however, the expression levels of *mokA*, *mokB*, and *mokH* were always higher in glutamic acid-containing medium than in original medium, and monacolin K production in glutamic acid medium was higher on day 11 than on day 8.

In glutamic acid-containing medium, *mokB, mokC, mokD, mokE, mokF, mokG, mokH,* and *mokI* levels were significantly upregulated. In contrast, *mokA* expression was decreased by 63.5% on day 8 of culture in the presence of glutamic acid compared with its expression in the original medium. Moreover, the expression levels of *mokB*, *mokC*, *mokD*, *mokE*, *mokF*, *mokG*, *mokH*,and *mokI*, which are thought to participate in monacolin K biosynthesis as structural genes (Wanping et al. 2015), were highest on day 8. This trend was similar to that observed for monacolin K production in glutamic acid-containing medium, indicating that glutamic acid stimulated monacolin K production via the upregulation of the expression levels of these eight genes. The expression levels of *mokA* were highest on day 11 in glutamic acid-containing medium, which differed from the trends observed for monacolin K production in glutamic acid medium. Although *mokA* is a key gene that encodes monacolin K polyketide synthase, our data showed that *mokA* was not essential for controlling the quantity of monacolin K production in glutamic acid.

Consistent with the results of the present study monacolin K production in *Monascus* was previously found to be positively influenced by glutamic acid. This tendency may be due to the expression of similar monacolin K biosynthetic genes in different strains or species. However, in this study, the highest monacolin K production by *Monascus* M1 was much higher than that observed in some other *Monascus* species. Moreover, upregulation of some monacolin K biosynthetic genes (i.e., *mokB*, *mokC*, *mokD*, *mokE*, *mokF*, *mokG*, *mokH*,and *mokI*) may be the major reason for the positive effects of glutamic acid on monacolin K production in *Monascus* M1. However, further comprehensive systematic analyses of the effects of glutamic acid on the genus *Monascus* are needed.

## Discussion

Monacolin K, also known as lovastatin, is able to act on cholesterol biosynthesis, which can reduce the function of HMG-CoA reductase as a competitive inhibitor. The monacolin K biosynthetic gene clusters in *Monascus* have received much attention because of the various biological activities of this compound. The monacolin K biosynthetic gene cluster has been identified according to the similarities with lovastatin synthetic genes (LNKS) in *Aspergillus*. Nine genes (*mokA*-*mokI*) have proposed the functions of these genes which were associated with the monacolin K synthesis. The *mokA*–deficient mutant in *M. pilosus* BCRC38072 cannot produce monacolin K, indicating that *mokA* encodes the polyketide synthase responsible for monacolin K biosynthesis in *M.pilosus* BCRC38072. Additionally, the *mokB*-deficient mutant of *M. pilosus* NBRC4480 cannot produce monacolin K, but exhibits accumulation of monacolin J, indicating that *mokB* is responsible for the synthesis of the diketide side chain of monacolin K (Sakai et al. 2009; Chen et al. 2015). Overexpression of the *mokH* gene in *M. pilosus* results in significantly higher monacolin K production than that in wild-type strains, indicating that *mokH* positively regulates monacolin K production (Chen et al. 2010). Based on previous reports, many factors, particularly nitrogen sources, such as amino acids, can influence the production of secondary metabolites in *Monascus*. For example, monacolin K production is increased in glutamic acid or leucine culture conditions; an ideal nitrogen source can be selected to control the low final pH and then produce citrinin-free *Monascus* pigments (Kang et al. 2013b). This is the first report on the inhibition of citrinin biosynthesis by controlling an extremely low pH. Previous also showed that lowering the pH value to 2.5 would result in high monacolin K and citrinin concentrations as well as high biomass in fixed dioscorea amount, implying that pH value may stimulate the formation of monacolin K and citrinin through increasing *Monascus* cell amount (Lee et al. 2007).

Previous studies have shown that the most suitable pH value for Monascus growth is about 4 (Lee et al. 2007). Interestingly, in this study, we found that the pH varied during different stages of growth; during the adjustment and logarithmic phases of *Monascus* growth, the pH was lower in glutamic acid-containig medium (4.68 and 4.88, respectively) than that of the original medium (5.15 and 6.02, respectively). Thus the amount of *Monascus* mycelia was greater in glutamic acid-containing medium than that in original medium. We hypothesized that glutamate may increase monacolin K production by increasing the density of *Monascus*.

In this study, we demonstrated, for the first time, the correlation between the expression levels of monacolin K biosynthetic genes and monacolin K production in *Monascus*. At any stage of cell growth, we found that glutamic acid enhanced monacolin K production in *Monascus* M1, compared with cultivation in original medium. When *Monascus* M1 was grown in glutamic acid-containing medium rather than original medium, monacolin K production increased from 48.4 to 215.4 mg l^−1^. Thus, these data showed that glutamic acid promoted the production of moncolinK.

*Monascus* expresses nine genes related to monacolin K synthesis, and monacolin K accumulation was found to be positively correlated with the expression of the monacolin K biosynthetic gene cluster. Indeed, RT-qPCR analysis showed that the maximal monacolin K biosynthesis quality was reached on day 8, at which point mostgenes related to monacolinK synthesis showed higher transcription in glutamic acid-containing medium, with the exception of *mokA*. These data indicated that *mokB*-*mokI* were eight key genes mediating monacolin K production in the presence of glutamic acid. According to the speculation on the function of monacolin K synthesis genes, we speculated that, *mokH* acts as a transcription factor, it will promoted the expression of key genes in monacolin K biosynthesis. *MokI* acts as an efflux pump, it will promoted the process of transferring monacolin K out of the *Monascus* cells, reduced the content of monacolin K in cells and promoted the final mknacolin K content. *MokB*-*mokG* participated in the monacolin K biosynthesis process and promoted the production of monacolin K in glutamic acid medium directly. Thus, based on the expression of monacolin K synthesis-related transcripts, these data supported that glutamic acid promoted the production of moncolin K has an internal power and the promoting effect is stable.

In summary glutamic acid increased the content of *Monascus* mycelia, altered the pH value in fermentation broth, changed the permeability of *Monascus* mycelia, enhanced the secretion of monacolin K to the outside of the cell, and reduced monacolin K content in the *Monascus* mycelia, thereby enhancing monacolin K production. In addition, glutamic acid may not only be used to provide energy for *Monascus* growth and metabolize, but also generate the production of acetyl coenzyme A, which is a substrate for monacolin K, and ultimately increase the content of metabolites. Our findings also demonstrated that glutamic acid could enhance monacolin K production by upregulating the expression of *mokB*, *mokC*, *mokD*, *mokE*, *mokF*, *mokG*, *mokH*,and *mokI*. So further studies are needed to elucidate the molecular pathways through which glutamic acid regulates monacolin K production.

## Abbreviations

LNKS: lovastatin synthetic genes
PDA: potato/dextrose/agar
HPLC: high-performance liquid chromatography
RT-qPCR: quantitative real-time reverse transcription polymerase chain reaction
SEM: scanning electron microscopy
PBS: phosphate-buffered sline
HMDS: hexamethyl disilazane
